# Sensitization of TRAIL-resistant LNCaP cells by resveratrol (3, 4', 5 tri-hydroxystilbene): molecular mechanisms and therapeutic potential

**DOI:** 10.1186/1750-2187-2-7

**Published:** 2007-08-24

**Authors:** Sharmila Shankar, Qinghe Chen, Imtiaz Siddiqui, Krishna Sarva, Rakesh K Srivastava

**Affiliations:** 1 Department of Biochemistry, University of Texas Health Science Center at Tyler, Tyler, Texas, USA 75703

## Abstract

**Background:**

We have previously shown that prostate cancer LNCaP cells are resistant to TRAIL, and downregulation of PI-3K/Akt pathway by molecular and pharmacological means sensitizes cells to undergo apoptosis by TRAIL and curcumin. The purpose of this study was to examine the molecular mechanisms by which resveratrol sensitized TRAIL-resistant LNCaP cells.

**Results:**

Resveratrol inhibited growth and induced apoptosis in androgen-dependent LNCaP cells, but had no effect on normal human prostate epithelial cells. Resveratrol upregulated the expression of Bax, Bak, PUMA, Noxa, Bim, TRAIL-R1/DR4 and TRAIL-R2/DR5, and downregulated the expression of Bcl-2, Bcl-X_L_, survivin and XIAP. Treatment of LNCaP cells with resveratrol resulted in generation of reactive oxygen species, translocation of Bax and p53 to mitochondria, subsequent drop in mitochondrial membrane potential, release of mitochondrial proteins (cytochrome c, AIF, Smac/DIABLO and Omi/HtrA2), activation of caspase-3 and caspase-9 and induction of apoptosis. The ability of resveratrol to sensitize TRAIL-resistant LNCaP cells was inhibited by dominant negative FADD, caspase-8 siRNA or N-acetyl cysteine. Smac siRNA inhibited resveratrol-induced apoptosis, whereas Smac N7 peptide induced apoptosis and enhanced the effectiveness of resveratrol.

**Conclusion:**

Resveratrol either alone or in combination with TRAIL or Smac can be used for the prevention and/or treatment of human prostate cancer.

## Background

Prostate cancer is the most frequently diagnosed male cancer and the second leading cause of cancer-related mortality in men in the Western world. In 2005, there were 232,090 new cases of prostate cancer diagnosed in the United States and 30,350 cancer deaths [1]. The molecular mechanisms responsible for the initiation and progression of prostate cancer have not been elucidated, and the only established risk factors for this disease include age, ethnic group, and family history [2]. In addition, the importance of diet in prostate carcinogenesis has been sown by epidemiological studies of Asian immigrants to Hawaii or California [3]. One of the most promising dietary compounds implicated in the chemoprevention of prostate cancer is resveratrol, a constituent of red wine and grapes [4-6].

Epidemiological and preclinical evidence suggests that polyphenolic phytochemicals possess cancer chemopreventive properties [4]. There is mounting evidence in the literature that resveratrol is a promising natural compound for prevention and treatment of a variety of human cancers [4-6]. Resveratrol (3,4',5 tri-hydroxystilbene) has been shown to possess a protective effect in prostate cancer in various pre-clinical animal models and has been reported to be effective in several other cancer types as well [7-10]. Numerous studies have reported interesting properties of resveratrol as a preventive agent against important pathologies i.e. vascular diseases, cancers, viral infection or neurodegenerative processes. Resveratrol acts on the process of carcinogenesis by affecting tumor initiation, promotion and progression, and suppresses the final steps of carcinogenesis, i.e. angiogenesis and metastasis. It induces apoptosis, and cell cycle arrest, and modulates several signal transduction pathways. Interestingly, resveratrol does not appear to be toxic in animal models. Furthermore, resveratrol has been shown to sensitize cancer cells to chemotherapy and radiotherapy. Despite considerable progress towards our understanding of the signaling pathways leading to resveratrol-mediated apoptosis, the molecular mechanisms by which resveratrol sensitizes prostate cancer cells to TRAIL treatment is not fully understood.

The tumor suppressor p53 protein plays a major role in cell cycle arrest, DNA repair and apoptosis [11]. It regulates apoptosis through both transcriptional-dependent and in-dependent mechanisms. Through transcription-dependent pathways, p53 functions as a transactivator to up-regulate downstream proapoptotic genes (e.g. Bax, Noxa, and PUMA), and functions as a repressor to down-regulate antiapoptotic genes (e.g. Bcl-2) promoting apoptosis. Bax induces apoptosis by enhancing the release of mitochondrial proteins (e.g. cytochrome c and Smac/DIABLO) to cytosol. Through transcription-independent pathways, p53 has a direct apoptogenic role where it translocates to mitochondria in response to cellular stress, resulting in apoptosis via interaction with antiapoptotic Bcl-2 and Bcl-X_L _proteins that alter the mitochondrial membrane potential and induce cytochrome *c *and Smac/DIABLO release into the cytosol with resultant caspase activation [12,13]. The elucidation of the p53-dependent pathway, resulting in mitochondrial outer membrane permeabilization through the pro-apoptotic Bcl-2 family proteins, is a key to unveiling the mechanism of stress-induced apoptosis. It is not know whether resveratrol regulates p53 functions via transcriptional independent mechanisms.

TRAIL (TNF-related apoptosis-inducing ligand) induces apoptosis in a wide variety of transformed cells [14-16]. It binds to several distinct receptors: (a) TRAIL-R1 (DR4) [17]; (b) TRAIL-R2 (DR5) [18]; (c) TRAIL-R3 (DcR1) [19]; and (d) TRAIL-R4 (DcR2) [20]. DR4 and DR5 contain the intracellular death domain (DD) which is essential for binding with an adaptor protein Fas-associated death domain (FADD) and the formation of active death-inducing signaling complex (DISC) [21,22]. In contrast, neither DcR1 nor DcR2 induce apoptosis due to a complete or partial lack of the intracellular DD, respectively [23,24]. TRAIL receptors are expressed in cancer cells but their expression may not be sufficient to induce apoptosis. The binding of TRAIL to DR4 and DR5 leads to the activation of caspase-8 or caspase-10 after recruitment of FADD [25,26], that in turn activates downstream effector caspases such as caspase-3, and caspase-7 [16]. Activation of caspase-8 or caspase-10 also cleaves BID (a Bcl-2 inhibitory protein) [27] to truncated BID (tBID), which triggers mitochondrial depolarization and causes subsequent release of mitochondrial proteins to cytosol [16,28-30]. Antiapoptotic Bcl-2 and Bcl-X_L _proteins preserve mitochondrial transmembrane potential and blocks the release of mitochondrial proteins such as cytochrome c, whereas Bax and Bak has opposite effect [31-33]. The cytochrome c binds to apaf-1 (apoptotic protease-activating factor 1) and, in the presence of dATP, recruits and activates procaspase-9 to form the apoptosome [30,34]. Inhibitor of apoptosis proteins (IAPs) interact with and inhibit caspase-3, caspase-7 and caspase-9. Finally, the activation of caspases causes cleavage of several substrates leading to apoptosis [30,34].

We and others have shown that TRAIL-resistant cancer cell lines can be sensitized by RNA synthesis inhibitors [35,36], protein synthesis inhibitors [35-37], chemotherapeutic agents [38-40], histone deacetylase inhibitors [41] or ionizing radiation [42,43]. Furthermore, we have recently demonstrated that downregulation of Akt/PKB or NFκB sensitized breast, prostate and lung cancer cells to TRAIL *in vitro *[36,37,44]. Study of the intracellular mechanisms that control TRAIL sensitivity may enhance our knowledge of death receptor-mediated signaling and help to develop resveratrol and/or TRAIL-based approaches to cancer prevention/treatment. Such information will not only allow rational design of resveratrol- and TRAIL-based strategies for prevention and/or treatment of prostate cancers but could also facilitate development of mechanism-driven combination protocols for optimal clinical effects. Elucidation of the mechanism(s) for anti-proliferative activity of resveratrol and TRAIL is an important step in a process aimed at ultimately applying this knowledge to clinical trials in humans. Prior to clinical trials, however, it is essential that the molecular mechanisms by which resveratrol sensitizes TRAIL-resistant cells are fully understood.

The objectives of our study were to examine the molecular mechanisms by which resveratrol sensitized TRAIL-resistant prostate cancer LNCaP cells. Here, we demonstrated that resveratrol inhibited cell growth, induced apoptosis and sensitized TRAIL-resistant LNCaP cells through multiple mechanisms. Resveratrol induced apoptosis through generating reactive oxygen species, targeting p53 to mitochondria, regulating Bcl-2 family members, death receptors DR4 and DR5) and IAPs, and releasing apoptogenic mitochondrial proteins (cytochrome c, Smac/DIABLO, and Omi/Htr2). Thus, resveratrol can be used either alone or in combination with TRAIL to prevent and/or treat human prostate cancer.

## Results

### Resveratrol sensitizes TRAIL-resistant prostate cancer LNCaP cells

**Figure 1:**
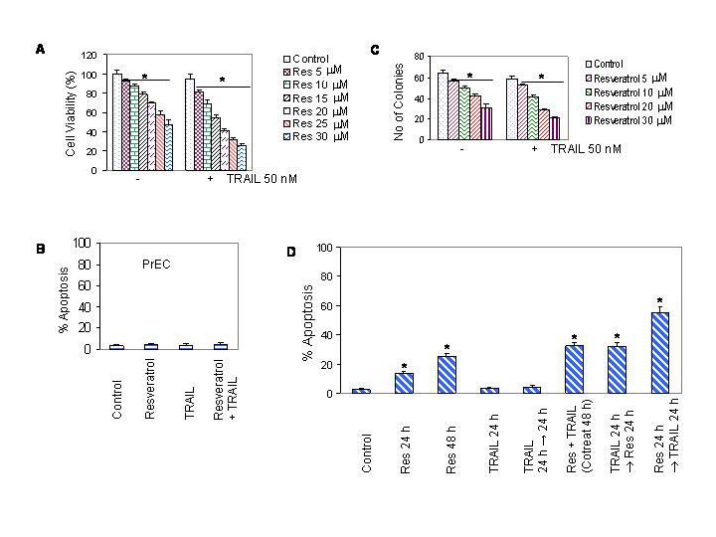
Interactive effects of resveratrol and TRAIL on cell viability, colony formation and apoptosis in prostate cancer cells. (A), Effects of resveratrol and/or TRAIL on cell viability in LNCaP cells. Cells were treated with various doses of resveratrol (0–30 μM) in the presence or absence of TRAIL (50 nM) for 48 h. Cell viability was measured by XTT assay as described in materials and methods. Data represent mean ± SD. * = Significantly different from respective control, P < 0.05. (B), Effects of resveratrol and/or TRAIL on apoptosis in human normal prostate epithelial cells (PrEC). PrEC were treated with resveratrol (20 μM) in the presence or absence of TRAIL (50 nM) for 48 h, and apoptosis was measured by TUNEL assay. (C), Effects of resveratrol and/or TRAIL on colony formation by LNCaP cells. Cells were treated with various doses of resveratrol (0–30 μM) in the presence or absence of TRAIL (50 nM). After three weeks, no of colonies were stained and counted. Data represent mean ± SD. * = Significantly different from respective control, P < 0.05. (D), Effects of different treatment combinations of resveratrol and TRAIL on apoptosis. LNCaP cells were treated with resveratrol (20 μM) in the presence or absence of TRAIL (50 nM). TRAIL was added simultaneously with resveratrol (cotreatment), before or after 24 h of resveratrol treatment. Apoptosis was measured by TUNEL assay. Data represent mean ± SD. * = Significantly different from respective control, P < 0.05.

We have previously shown that TRAIL induces apoptosis in prostate cancer cells with varying sensitivity, and LNCaP cells are resistant to TRAIL [36]. We first measured the effects of resveratrol and/or TRAIL on cell viability of LNCaP cells expressing wild type p53 (Fig. 1A). Resveratrol (10–30 μM) inhibited cell viability in LNCaP cells in a dose-dependent manner (Fig. 1A). By comparison, TRAIL had no effects in LNCaP cells. Interestingly, resveratrol sensitized TRAIL-resistant LNCaP cells. We next examined whether resveratrol and/or TRAIL had any effect on human normal prostate epithelial cells (PrEC) (Fig. 1B). Resveratrol in the presence or absence of TRAIL had no effect on apoptosis in human normal prostate epithelial cells.

Since anchorage independent growth is one of the characteristics of tumor formation, we sought to measure the effects of resveratrol and TRAIL on colony formation in soft agar (Fig. 1C). Resveratrol inhibited colony growth of LNCaP cells in a dose-dependent manner. By comparison, TRAIL had no effects on colonies formed by LNCaP cells. Resveratrol sensitized LNCaP colonies to TRAIL treatment. These data demonstrate that resveratrol inhibits anchorage-dependent and independent growth of prostate cancer cells, and enhances the therapeutic potential of TRAIL.

The sequence of drug administration is important to obtain maximum therapeutic benefits in combination therapy. The pretreatment of cells with resveratrol may increase the apoptotic effects of TRAIL by up-regulating death receptors. We therefore examined whether pretreatment of LNCaP cells with resveratrol followed by TRAIL induced more apoptosis than the concurrent treatment or TRAIL followed by resveratrol (Fig. 1D). LNCaP cells were pretreated with resveratrol for 24 h, followed by TRAIL for another 24 h, and vice versa. Interestingly, the pretreatment of cells with resveratrol followed by TRAIL induced more apoptosis than concurrent treatment or single agent alone. In order to understand this synergistic interaction, reverse sequence of drug exposure was used in which cells were pretreated with TRAIL for 24 h followed by treatment with resveratrol for additional 24 h. Reverse sequence of treatment, TRAIL followed by resveratrol, has resulted significantly less apoptosis than the sequential treatment of cells with resveratrol followed by TRAIL. However, there was no difference between concurrent treatment and TRAIL followed by resveratrol. These data suggest that sequential treatment of cells with resveratrol followed by TRAIL can be used to enhance the apoptosis inducing potential of TRAIL.

### Resveratrol upregulates TRAIL-R1/DR4 and TRAIL-R2/DR5 receptors

**Figure 2:**
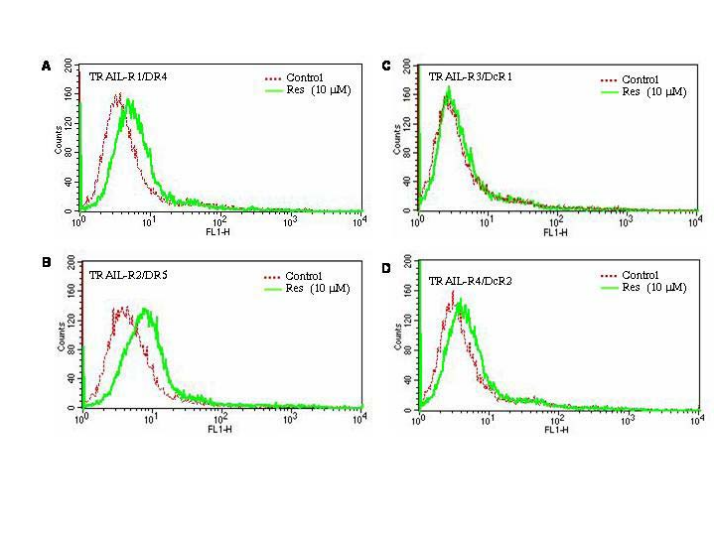
Effects of resveratrol on the surface expression of death receptors. (A, B, C and D), LNCaP cells were treated with resveratrol (0, or 10 μM) for 48 h and expressions of death receptors TRAIL-R1/DR4, TRAIL-R2/DR5, TRAIL-R3/DcR1 and TRAIL-R4/DcR2 were measured by flowcytometry.

We and others have shown that TRAIL induces apoptosis by binding to TRAIL-R1/DR4 and TRAIL-R2/DR5 [25,26]. Upregulation of death receptor on the cell-surface may either enhance the biological activity of TRAIL in sensitive cells or sensitize TRAIL-resistant cells. Since resveratrol enhances the apoptosis-inducing potential of TRAIL, we sought to examine the effects of resveratrol on death receptor expressions in LNCaP cells. Resveratrol induced expression of death receptors TRAIL-R1/DR4 and TRAIL-R2/DR5 in LNCaP cells (Fig. 2A and 2B). In contrast, it has no effect on the expression of decoy receptors DcR1 and DcR2 (Fig. 2C and 2D). These data suggest that upregulation of death receptors DR4 and/or DR5 may be one of the mechanisms by which resveratrol sensitizes prostate cancer cells to TRAIL.

### Resveratrol sensitizes TRAIL-resistant LNCaP cells through activation of caspases and cleavage of Poly-ADP Ribose Polymerase (PARP)

**Figure 3:**
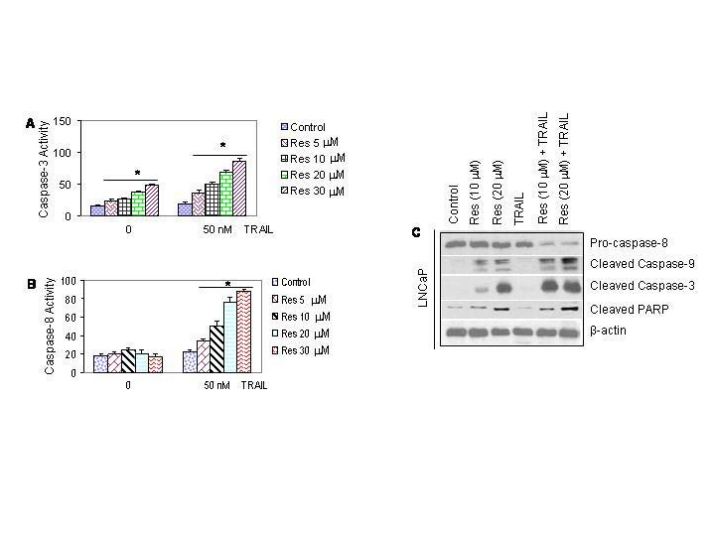
Interactive effects of resveratrol and TRAIL on caspase activation and PARP cleavage. (A), Effects of resveratrol and/or TRAIL on caspase-3 activity in LNCaP cells. Cells were treated with resveratrol (0–30 μM) in the presence or absence of TRAIL (50 nM) for 24 h. At the end of incubation period, caspase-3 activity was measured by flurometric assay. (B), Effects of resveratrol and/or TRAIL on caspase-8 activity. LNCaP cells were treated with resveratrol (0–30 μM) in the presence or absence of TRAIL (50 nM) for 24 h. At the end of incubation period, caspase-8 activity was measured by flurometric assay. (C), Effects of resveratrol and/or TRAIL on cleavage of pro-caspase-8, pro-caspase-3, pro-caspase-9 and PARP. LNCaP cells were pretreated with resveratrol (0, 10 or 20 μM) for 24 h followed by treatment with or without TRAIL (50 nM) for 24 h. At the end of incubation period, cells were harvested, and the Western blot analysis was performed to measure the expression of pro-caspase-8, cleaved-caspase-3, cleaved-caspase-9 and PARP. β-actin was used as a loading control.

Caspase activation plays an important role in apoptosis triggered by stress stimuli. We therefore examined the activation of caspase-3 and caspase-8 by fluorogenic assay and Western blot analysis in LNCaP cells (Fig. 3A–C). Resveratrol induced caspase-3 activity in LNCaP cells in a dose-dependent manner (Fig. 3A). TRAIL had no effects on caspase-3 activity (Fig. 3A and 3B). Resveratrol and TRAIL alone had no significant effect on caspase-8 activation (Fig. 3B). However, the combination of resveratrol and TRAIL enhanced caspase-3 and caspase-8 activities.

Poly-ADP Ribose Polymerase (PARP) is one of the caspase substrates which is cleaved by caspase(s) [25,41-43]. We therefore examined the cleavage of pro-caspase-3, pro-caspase-8, pro-caspase-9, and PARP in LNCaP cells by the Western blot analysis (Fig. 3C). Caspase-8 antibody recognizes the whole molecule, whereas caspase-9, caspase-3 and PARP antibodies recognize their cleavage products. Treatment of LNCaP cells with resveratrol resulted in cleavage of pro-caspase-3, and pro-caspases-9, but has effect on cleavage of pro-caspase-8. TRAIL alone had no effect on the cleavage of pro-caspase-8, pro-caspase-3, pro-caspase-9 and PARP compared to untreated control. Interestingly, the combination of resveratrol and TRAIL significantly cleaved pro-caspase-8, pro-caspase-9, pro-caspase-3 and PARP.

### Resveratrol regulates expression of Bcl-2 family members and inhibitors of apoptosis proteins (IAPs)

**Figure 4:**
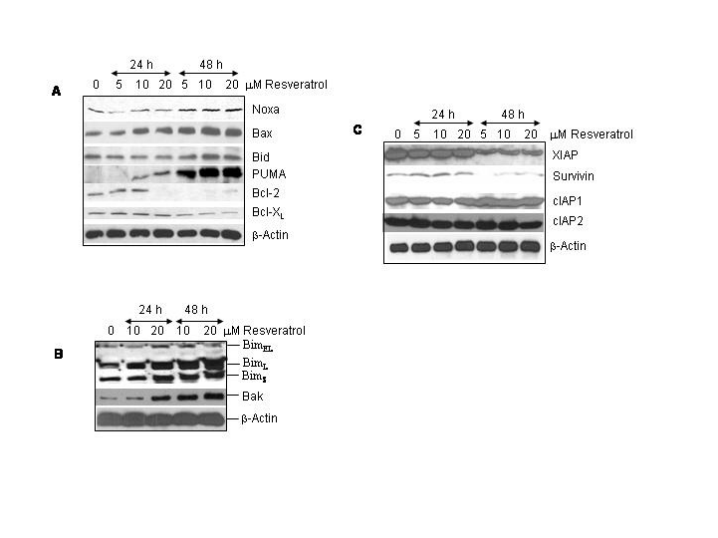
Effects of resveratrol on the expressions of Bcl-2 family members and IAPs. (A and B), Effects of resveratrol on protein expression of Bcl-2 family members. Cells were treated with resveratrol (0–20 μM) for 24 or 48 h. The expressions of Noxa, Bim, Bak, Bax, Bid, PUMA, Bcl-2, and Bcl-X_L _were examined by Western blot analysis. Actin antibody was used as a loading control. Bim_EL _= Bim extra large, Bim_L _= Bim large, Bim_S _= Bim short. (C), Effects of resveratrol on the expressions of IAPs. Cells were treated with resveratrol (0–20 μM) for 24 or 48 h. Crude proteins were subjected to SDS-PAGE and immunoblotted with antibody specific for XIAP, survivin, cIAP1 or cIAP2. β-actin antibody was used as a loading control.

Bcl-2 family members are important regulator of apoptosis, and overexpression of Bcl-2 and Bcl-X_L _may confer resistance to chemotherapy [31,32]. They regulate mitochondrial outer membrane integrity, cytochrome c release, caspase activation and apoptosis [45-47]. Some of the proteins within this family, including Bcl-2 and Bcl-X_L _inhibit apoptosis, and others, such as Bax, Bak, Noxa and Bim promote apoptosis. We therefore sought to examine the effects of resveratrol on Bcl-2 family members in LNCaP cells (Fig. 4A and 4B). Resveratrol induced expression of proapoptotic proteins Noxa, Bim, Bak, Bak and Bid, and inhibited antiapoptotic proteins Bcl-2 and Bcl-X_L_. These data suggest that resveratrol regulates both pro- and anti-apoptotic members of Bcl-2 family.

Inhibitors of apoptosis proteins (IAPS) have been shown to neutralize the activity of caspases [48]. We therefore examined the effects of resveratrol on the expression of XIAP, survivin, cIAP1 and cIAP2 (Fig. 4C). None of the doses of resveratrol inhibited the expression of IAPs at 24 h. However, resveratrol inhibited the expression of XIAP and survivin at 48 h. There was no effect of resveratrol on cIAP1 and cIAP2 at 48 h. These data suggest that inhibition of XIAP and survivin by resveratrol may sequester less caspase(s) and thus enhance apoptosis induction by resveratrol.

### Resveratrol dysrupts mitochondrial homeostasis and causes the release of mitochondrial proteins

**Figure 5:**
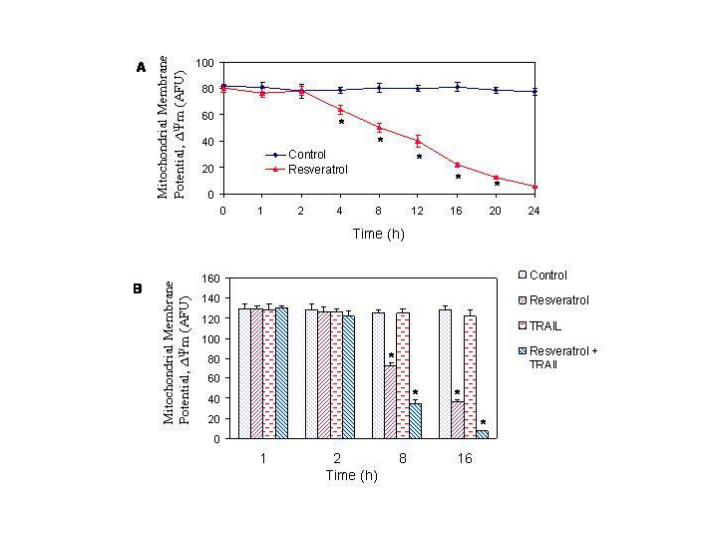
Effects of resveratrol and/or TRAIL on mitochondrial membrane potential (Δψ_m_). (A), Resveratrol induces drop in Δψ_m_. LNCaP cells were treated with or without resveratrol (20 μM) for 0–24 h. Cells were stained with JC1 dye, and Δψ_m _was measured by a fluorometer as per manufacturer's instructions. (B), Interactive effects of resveratrol and TRAIL on Δψ_m_. LNCaP cells were treated with resveratrol (20 μM) in the presence or absence of TRAIL (50 nM) for 1, 2, 8 and 16 h. Cells were stained with JC1 dye, and Δψ_m _was measured.

During apoptosis, engagement of the mitochondrial pathway involves the permeabilization of the outer mitochondrial membrane (OMM), which leads to the release of cytochrome c and other apoptogenic proteins [49,50]. OMM permeabilization depends on activation, translocation and oligomerization of multidomain Bcl-2 family proteins such as Bax. We therefore measured mitochondrial membrane potential (Δψ_m_), and release of cytochrome c, AIF, Smac/DIABLO, and Omi/HtrA2 from mitochondria to cytosol. Treatment of prostate cancer LNCaP cells with resveratrol resulted in a drop off Δψ_m _over time (Fig. 5A). Since resveratrol sensitized TRAIL-resistant cells to undergo apoptosis, we next examined the interactive effects of resveratrol and TRAIL on Δψ_m _(Fig. 5B). Resveratrol and TRAIL alone or in combination with each other had no effect on Δψ_m _up to 2 h. Although TRAIL was ineffective alone, the treatment of LNCaP cells with resveratrol caused a drop in Δψ_m _at 8 h and 16 h. Interestingly, the combination of resveratrol and TRAIL resulted in significant drop in Δψ_m _at both time points.

**Figure 6:**
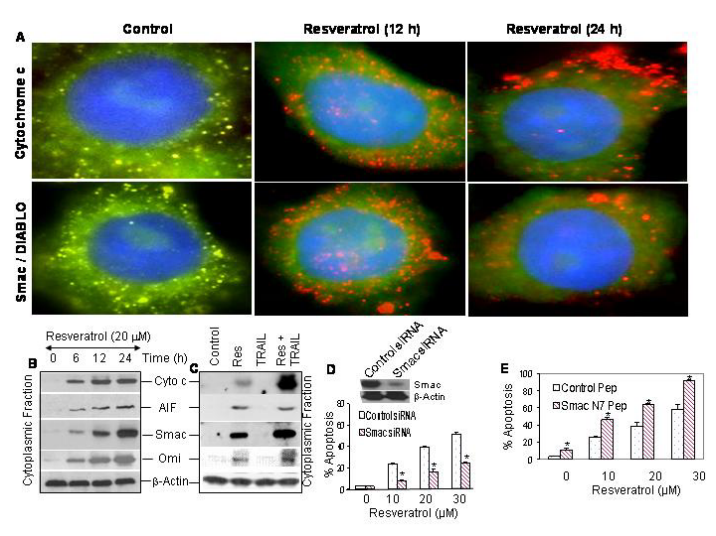
Effects of resveratrol and/or TRAIL on mitochondrial dysfunction. (A), Resveratrol induces the release of cytochrome c and Smac/DIABLO from mitochondria to cytosol. LNCaP cells were treated with or without resveratrol (20 μM) for 12 or 24 h. Cells were fixed, permeabilized and stained with anti-cytochrome c or anti-Smac/DIABLO antibody at 4°C for 18 h. After washing, cells were stained with mitotracker red (mitochondrial staining), DAPI (nuclear staining) and secondary antibody conjugated with FITC (for cyto c or Smac). Red color = mitochondria, green color = cytochrome c or Smac/DIABLO, blue color = nucleus, yellow = co-locolization of cyto c and Smac/DIABLO to mitochondria. (B), Resveratrol releases mitochondrial proteins. LNCaP cells were treated with resveratrol (20 μM) for 0, 6, 12, or 24 h, and cytoplasmic fractions were prepared. Crude proteins were subjected to SDS-PAGE and immunoblotted with anti-cytochrome c (Cyto C), anti-AIF, anti-Smac/DIABLO or anti-Omi antibody. β-Actin was used as a loading control. (C), Interactive effects of resveratrol and TRAIL on the release of mitochondrial proteins. LNCaP cells were treated with resveratrol (20 μM) in the presence or absence of TRAIL (50 nM) for 12 h, and cytoplasmic fractions were prepared. Crude proteins were subjected to SDS-PAGE and immunoblotted with anti-Cyto C, anti-AIF, anti-Smac/DIABLO or anti-Omi antibody. β-Actin was used as a loading control. (D), Effects of Smac siRNA on resveratrol-induced apoptosis. Western blotting data demonstrate that transient transfection of LNCaP cells with Smac siRNA plasmid inhibited Smac protein expression at 48 h. LNCaP cells were transiently transfected with either control plasmid or plasmid expressing Smac siRNA along with plasmid (pCMV-LacZ) encoding the β-galactosidase (β-*Gal*) enzyme. There was no difference in transfection efficiency between groups. Cells were treated with various doses of resveratrol (0–30 μM) for 48 h, and apoptosis was measured. (E), Enhancement of resveratrol-induced apoptosis by Smac N-7 peptide. LNCaP cells were pretreated with either 25 μM control Smac peptide or Smac N7 peptide for 4 h, and treated with various doses of resveratrol (10, 20 or 30 μM) for 48 h. Apoptosis was measured by TUNEL assay. Data represent mean ± SD. * = significantly different from respective control (P < 0.05).

We next examined the effects of resveratrol on the release of cytochrome c and Smac/DIABLO from mitochondria to cytosol by immunocytochemistry (Fig. 6A). In control cells, cytochrome c and Smac were predominantly in mitochondria as evident by yellow color mitochondria (colocalization of green color cyto c or Smac in red color mitochondria). Treatment of LNCaP cells with resveratrol resulted in the release of cytochrome c and Smac from mitochondria to cytosol (appearance of red color mitochondria, and diffused green color of cyto c or Smac).

It has been shown that mitochondrial proteins such as cytochrome c, AIF, Smac/DIABLO and Omi/Htr2 are released into the cytosol in cells undergoing apoptosis [51,52]. These proteins are involved in caspase-dependent and -independent apoptosis. We next confirmed the release of mitochondrial proteins in LNCaP cells treated with resveratrol in the presence of absence of TRAIL by Western blotting using cytoplasmic fraction (Fig. 6B and 6C). Treatment of LNCaP cells with resveratrol caused the release of cytochrome c, AIF, Smac/DIABLO and Omi/Htr2 from mitochondria to cytosol in a time-dependent manner. TRAIL had no effect on the release of mitochondrial proteins. The combination of resveratrol and TRAIL enhanced the release of cytochrome c, AIF and Omi from mitochondria to cytosol.

We next confirmed the involvement of Smac/DIABLO in resveratrol-induced apoptosis by inhibition of Smac expression (RNAi technology) or exogenous administration of Smac peptide (Fig. 6D and 6E). We have previously shown that Smac N7 peptide can sensitize resistant cells to undergo apoptosis by TRAIL [28] or curcumin [53]. The data revealed that plasmid expressing Smac siRNA significantly inhibited resveratrol-induced apoptosis (Fig. 6D), and Smac N7 peptide significantly enhanced apoptosis induced by resveratrol (Fig. 6E). These data suggest that Smac is an important regulator of resveratrol-induced apoptosis.

### Resveratrol induces Bax and p53 translocation to mitochondria

**Figure 7:**
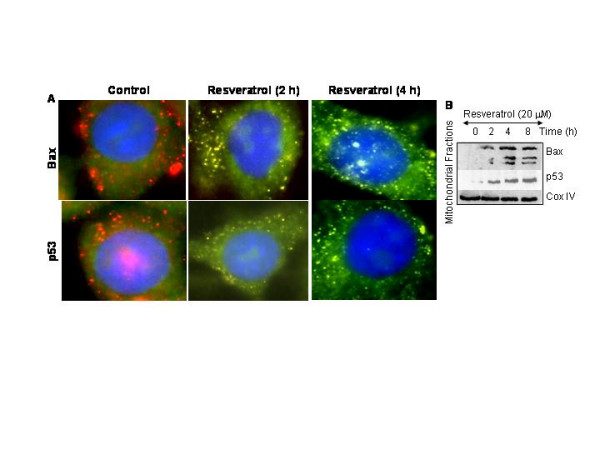
Translocation of Bax and p53 to mitochondria. (A), Resveratrol induces translocation of Bax and p53 to the mitochondria. LNCaP cells were treated with or without resveratrol (20 μM) for 2 or 4 h. Cells were fixed, permeabilized and stained with anti-Bax antibody at 4°C for 18 h. After washing, cells were stained with mitotracker red (mitochondrial staining), DAPI (nuclear staining) and secondary antibody conjugated with FITC (for Bax). Red color = mitochondria, green color = Bax, blue color = nucleus, yellow = translocation of Bax or p53 to mitochondria. (B), Translocation of Bax and p53 to mitochondria. LNCaP cells were treated with resveratrol for 0, 2, 4 or 8 h. Mitochondrial fractions were prepared, subjected to SDS-PAGE, and immunoblotted with anti-Bax, anti-p53 or anti-COX IV antibody.

The proapoptotic protein Bax is translocated to mitochondria where it oligomerizes with Bak and causes the release of mitochondrial proteins [28,54]. Furthermore, a new role of p53, a transcriptional independent mechanism, has recently been reported where it translocates to mitochondria and interacts with the Bcl-2 family members to regulate apoptosis. We therefore examined whether resveratrol induces translocation of Bax and p53 to mitochondria by immunohistochemistry (Fig. 7A) and Western blotting of mitochondrial fractions (Fig. 7B). We have used anti-Bax 6A7 antibody (BD Pharmingen) which recognizes the conformationally altered Bax protein. In control cells, Bax and p53 were not colocalized to mitochondria which was clear from distinct red (mitochondria) and green (Bax or p53) color. Treatment of LNCaP cells with resveratrol resulted in translocation of Bax and p53 to mitochondria (appearance of yellow mitochondria) at 2 and 4 h. We next confirmed the translocation of Bax and p53 by Western blot analysis in isolated mitochondrial fraction (Fig. 7B). Treatment of LNCaP cells with resveratrol resulted in translocation of Bax and p53 to mitochondria as early as 2 h and continued up to 8 h. The Bax antibody recognizes three bands which could be monomer, dimmer and tetramer of Bax. Interestingly, translocation of Bax and p53 to the mitochondria (2 h) preceded the drop in mitochondrial membrane potential and cytochrome c release (6 h).

### Death receptor pathway is involved in sensitization of TRAIL-resistant LNCaP cells by resveratrol

**Figure 8:**
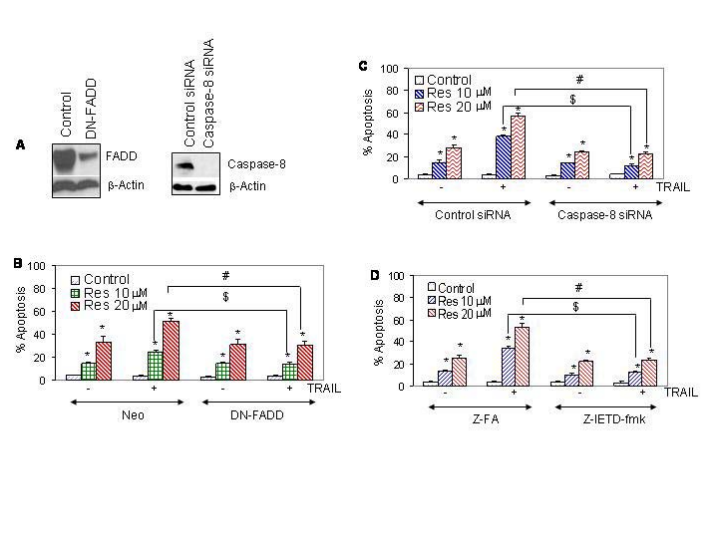
Involvement of death receptor pathway. (A), Expression of FADD and caspase-8 by Western blot analysis. LNCaP cells were transiently transfected with either control plasmid or plasmid expressing dominant negative FADD (DN-FADD) or caspase-8 siRNA along with plasmid (pCMV-LacZ) encoding β-galactosidase (β-*Gal*) enzyme. There was no difference in transfection efficiency among groups. Cell lysates were run on SDS-PAGE to measure the expression of FADD and caspase-8 by Western blot analysis. (B), Effects of dominant negative FADD on resveratrol and/or TRAIL-induced apoptosis. LNCaP cells were transiently transfected as described above. Cells were treated with resveratrol (0, 10 or 20 μM) in the presence or absence of TRAIL (50 nM) for 48 h, and apoptosis was measured. Data represent mean ± SD. * = Significantly different from respective control; # and $ = treatment groups were significantly different, P < 0.05. (C), Effects of caspase-8 siRNA on resveratrol and/or TRAIL-induced apoptosis. LNCaP cells were transiently transfected as described above. Cells were treated with resveratrol (0, 10 or 20 μM) in the presence or absence of TRAIL (50 nM) for 48 h. Apoptosis was measured by DAPI staining. Data represent mean ± SD. * = Significantly different from respective control; # and $ = treatment groups were significantly different, P < 0.05. (D), Effects of caspase-8 inhibitors on resveratrol and/or TRAIL-induced apoptosis. LNCaP cells were pretreated with either control peptide or caspase-8 inhibitor z-IETD-fmk (50 μM) for 4 h, followed by treatment with resveratrol (0, 10 or 20 μM) in the presence or absence of TRAIL (50 nM) for 48 h. Apoptosis was measured by DAPI staining. Data represent mean ± SD. * = Significantly different from respective control; # and $ = treatment groups were significantly different, P < 0.05.

Since pretreatment of LNCaP cells with resveratrol sensitizes TRAIL-resistant cells to undergo apoptosis, we sought to examine the contribution of death receptor pathway by using dominant negative FADD and caspase-8 siRNA. The FADD is an adapter protein which links death receptors to procaspases-8 in cells undergoing apoptosis [22]. In order to examine the involvement of death receptor pathway, we inhibited the expression of FADD or caspase-8. LNCaP cells were transfected with plasmid expressing DN-FADD, caspase-8 siRNA or respective controls (Fig. 8A). Data revealed that DN-FADD and caspase-8 siRNA inhibited the expression of FADD and caspase-8 proteins, respectively. We next examined whether overexpression of DN-FADD or caspase-8 siRNA inhibited the ability of resveratrol to sensitize cells to TRAIL (Fig. 8B and 8C). Resveratrol induced apoptosis in a dose-dependent manner, whereas TRAIL had no effect. Pretreatment of LNCaP cells with resveratrol sensitized cells to undergo apoptosis by TRAIL. As expected, DN-FADD or caspase-8 siRNA inhibited the ability of resveratrol to sensitize LNCaP cells to TRAIL. We also confirmed the role of caspase-8 by using the caspase-8 inhibitor z-IETD-fmk. Caspase-8 inhibitor also inhibited the ability of resveratrol to sensitize TRAIL-resistant cells (Fig. 8D). These data suggest the involvement of death receptor pathway in sensitization of TRAIL-resistant cells by resveratrol.

### Sensitization of TRAIL-resistant LNCaP cells by resveratrol involves ROS production

**Figure 9:**
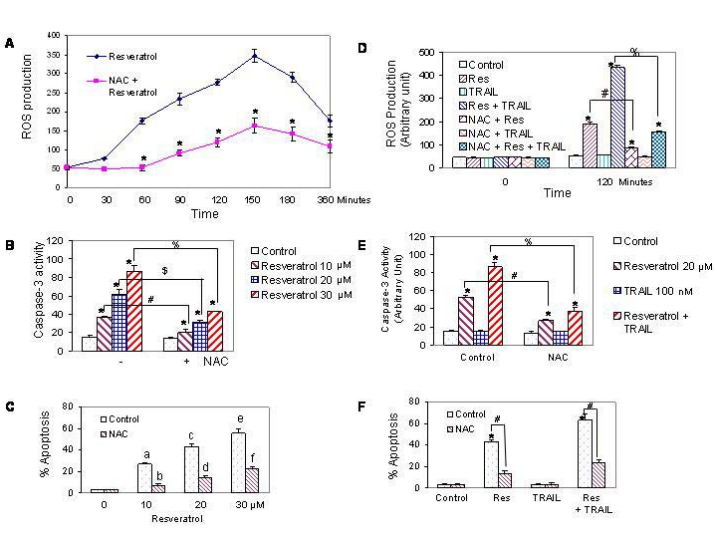
Involvement of reactive oxygen species in sensitization of TRAIL-resistant LNCaP cells by resveratrol. (A), Generation of ROS by resveratrol. LNCaP cells were seeded in 96-well plates, loaded with 5 μM CM-H_2_DCFDA dye for 30 min, and treated with either resveratrol (20 μM) or N-acetylcysteine (NAC) (50 mM) plus resveratrol (20 μM) for 0–360 min. Fluorescence was measured by a fluorometer as per manufacturer's instructions (EMD Biosciences/Molecular Probes). * = significantly different from respective controls, P < 0.05. (B), Inhibition of resveratrol-induced caspase-3 activity by NAC. LNCaP cells were pretreated with 50 mM NAC for 2 h followed by treatment with resveratrol (10, 20 or 30 μM) for 12 h, and caspase-3 activity was measured by a fluorometer as per manufacturer's instructions. * = significantly different from respective control; %, # or $ = significantly different from each other, P < 0.05. (C), Inhibition of resveratrol-induced apoptosis by NAC. LNCaP cells were pretreated with 50 mM NAC for 2 h followed by treatment with resveratrol (10, 20 or 30 μM) for 48 h, and apoptosis was measured by TUNEL assay. a, c and e were significantly different from b, d and f, respectively, P < 0.05. (D), Interactive effects of resveratrol and TRAIL on ROS production. LNCaP cells were pretreated with NAC (50 mM) for 2 h followed by treatment with resveratrol (20 μM) with or without TRAIL (50 nM) for 120 min., and ROS production was measured. * = significantly different from respective controls, P < 0.05; # or % = significantly different between groups, P < 0.05. (E), Interactive effects of resveratrol and TRAIL on caspase-3 activity. LNCaP cells were pretreated with 50 mM NAC for 2 h followed by treatment with resveratrol (20 μM) for 12 h, and caspase-3 activity was measured. * = significantly different from respective controls, P < 0.05; # or % = significantly different between groups, P < 0.05. (F), Interactive effects of resveratrol and TRAIL on apoptosis. LNCaP cells were pretreated with 50 mM NAC for 2 h followed by treatment with resveratrol (20 μM) with or without TRAIL (50 nM) for 48 h, and apoptosis was measured by TUNEL assay. * = significantly different from respective controls, P < 0.05; # = significantly different between groups, P < 0.05.

Recent studies have shown that cancer preventive agent induced apoptosis through generation of ROS. Generation of reactive oxygen species by oxidative damage play an important role in cells undergoing apoptosis [30,55]. The treatment of LNCaP cells with resveratrol resulted in ROS production with a maximum at 150 min, after that it slightly declined (Fig. 9A). We next examined whether, resveratrol causes oxidative stress that is responsible for caspase-3 activation and apoptosis. Pretreatment of LNCaP cells with 50 mM N-acetylcysteine (NAC) followed by resveratrol (0–30 μM) resulted in a significant inhibition of resveratrol-induced caspase-3 activity (Fig. 9B) and apoptosis (Fig. 9C). Taken together, our results suggest that resveratrol-induced apoptosis is also mediated through generation of ROS, which, in turn, may activate cell intrinsic pathway of apoptosis.

Since resveratrol sensitizes TRAIL-resistant LNCaP cells to undergo apoptosis, we measured the involvement of ROS in this process. As shown above, treatment of LNCaP cells with resveratrol resulted in generation of ROS, activation of caspase-3, and induction of apoptosis (Fig. 9D, E and 9F). TRAIL alone had no effect on ROS production, although it induced caspase-3 activity and apoptosis. The combination of resveratrol and TRAIL significantly produced more ROS than resveratrol alone (Fig. 9A). The pretreatment of LNCaP cells with NAC inhibited generation of ROS by resveratrol alone, and resveratrol and TRAIL. Similarly, the pretreatment of LNCaP cells with NAC inhibited caspase-3 activity and apoptosis induced by resveratrol alone, and resveratrol and TRAIL. These data suggest that generation of ROS is one of the mechanisms by which resveratrol sensitizes TRAIL-resistant LNCaP cells.

## Discussion

Dietary supplements and complementary and alternative medication (CAM) are becoming increasingly popular among patients with cancer. Recently, there has been major interest in development of compounds of natural origin with chemopreventive and chemotherapeutic properties. Resveratrol is one such compound that is found in high concentrations in red wine and has a wide range of promising pharmacological properties. In the present study, we have shown that resveratrol not only induced apoptosis but also sensitized TRAIL-resistant human prostate cancer LNCaP cells through activation of multiple signaling pathways. Resveratrol-induced apoptosis disrupted mitochondrial homeostasis which was evident by the drop in mitochondrial membrane potential and release of mitochondrial proteins (cytochrome C, Smac/DIABLO, AIF, and Omi/HtrA2). Resveratrol induced expression of proapoptotic proteins (Bax, Bak, PUMA, Noxa and Bim) and inhibited expression of antiapoptotic proteins (Bcl-2 and Bcl-X_L_). These proteins exert their effect mainly at the level of mitochondria. Furthermore, resveratrol generated ROS, translocated p53 and Bax to mitochondria where these proteins may interact with other Bcl-2 family members to cause permeabilization of outer mitochondrial membrane and release of mitochondrial proteins leading to caspase activation and apoptosis. Finally, the ability of resveratrol to sensitize TRAIL-resistant cells suggest that its can be used either alone or in combination with TRAIL to prevent and/or treat human prostate cancer.

Apoptosis can be initiated by extracellular and intracellular signals that trigger a complex machinery of pro-apoptotic proteases and mitochondrial functions. It is integration of multiple survival and death signals that determine whether a cell is to survive or undergo apoptosis. We and others have shown that the Bcl-2 family members are the mediators of cell survival and apoptosis [31,32,45,51]. The inhibition of antiapoptotic Bcl-2 and Bcl-X_L_, and induction of proapoptotic Bax, Bak, PUMA, Noxa, Bim, and Bid proteins by resveratrol suggest that the relative expression of these proteins can determine the sensitivity of cancer cells to TRAIL. In a recent report, we have shown that another chemopreventive agent curcumin inhibited the expression of antiapoptotic Bcl-2 and Bcl-X_L_, and induced the expression of proapoptotic Bax, Bak, PUMA, Noxa, and Bim in prostate cancer cells [53]. Similarly, resveratrol enhanced the apoptosis-inducing potential of TRAIL in androgen-insensitive prostate cancer PC-3 cells by regulating Bcl-2 family members [56].

In the present study, resveratrol induced the release of mitochondrial Smac/DIABLO, cytochrome c, AIF and Omi/HtrA2. Cytochrome c forms apoptosomes, in the presence of ATP/dATP and Apaf-1, which activate caspase-9 and subsequently effector caspases such as caspase-3 and caspase-7 leading to cell death [34]. Smac/DIABLO, AIF and Omi do not require apoptosome formation for apoptosis induction. In comparison, Smac/DIABLO binds to IAPs (XIAP, survivin, c-IAP-1, or c-IAP-2) and abrogates IAP-mediated inhibition of caspase-3 and caspase-7, thereby facilitating caspase-mediated apoptosis. The pro- and anti-apoptotic Bcl-2 family proteins regulate the release of these mitochondrial proteins. Furthermore, our data demonstrate that resveratrol inhibits the expression of XIAP and survivin, suggesting the inhibition of IAPs will allow more free caspases to bind their substrates and finally induce apoptosis.

We have previously demonstrated that TRAIL induces apoptosis in prostate cancer cells through the release of cytochrome c and Smac/DIABLO [40,43], and Bax and Bak differentially regulate the release of cytochrome c and Smac/DIABLO from mitochondria to cytosol in MEFs [28]. Here, we showed that TRAIL-resistant LNCaP cells can be sensitized not only by resveratrol, but also by the mature form of Smac/DIABLO which will bypass mitochondria. Our data clearly demonstrate the requirement of Smac/DIABLO for sensitization of TRAIL-resistant LNCaP cells. We have demonstrated that the down-regulation of Smac by RNAi conferred resistance whereas addition of a Smac-mimetic peptide enhanced resveratrol-induced apoptosis, suggesting that Smac release is required for efficient resveratrol-induced apoptosis. To our knowledge, this represents the first finding that regulation of Smac release by resveratrol is an important event for apoptosis induction. However, Smac RNAi could not completely suppress resveratrol-induced apoptosis, suggesting that there may be additional, Smac-independent, mechanisms operating to induce apoptosis. In support of this, our data suggest that other mitochondrial proteins cytochrome c, AIF and Omi/HtrA2 may contribute cell death process.

TP53 is the most frequently mutated and intensely studied tumor suppressor gene [57,58]. After DNA damage or proto-oncogene activation, p53 is stabilized by post-translational modifications and exerts its anti-tumorigenic activity by inducing cell cycle arrest or apoptosis [59,60]. Although most data suggest that p53 regulates its activities through transcription activation, there is growing evidence that p53 exerts pro-apoptotic activity in a transcription-independent manner [61]. In response to appropriate apoptotic signals, p53 translocates to mitochondria, where it interacts with Bak, Bcl-2 and Bcl-X_L _and induces the release of mitochondrial proteins (e.g. cytochrome c) by acting in a manner similar to the BH-3-only pro-apoptotic proteins [62,63]. Caspase activation by p53 occurs through the release of apoptogenic factors from the mitochondria, including cytochrome c and Smac/DIABLO. Similarly, we have shown that curcumin induced translocation of p53 to mitochondria and disrupted mitochondrial homeostasis in prostate cancer cells [53]. In the present study, resveratrol induced translocation of p53 to mitochondria as early as 2 h, and the time of translocation of p53 was similar to that of proapoptotic protein Bax.

Reactive oxygen species (ROS) include oxygen ions, free radicals and peroxides both inorganic and organic. They are generally very small molecules and are highly reactive due to the presence of unpaired valence shell electrons. ROS are formed as natural byproduct of the normal metabolism of oxygen and have important roles in cell signaling [64]. However, during times of environmental stress ROS levels can increase dramatically, which can result in significant damage to cell structures. This cumulates into a situation known as oxidative stress. Cells are normally able to defend themselves against ROS damage through the use of enzymes such as superoxide dismutases and catalases. Small molecule antioxidants such as ascorbic acid (vitamin-C), uric acid, and glutathione also play important roles as cellular antioxidants. Similarly, polyphenol antioxidants assist in preventing ROS damage by scavenging free radicals. At low levels, ROS such as superoxide, hydrogen peroxide, and hydroxyl radical, may function in cell signaling processes, whereas at higher levels, ROS may damage cellular macromolecules (such as DNA and RNA) and participate in apoptosis [64,65]. ROS have been implicated as a key factor in the activation of p53 by many chemotherapeutic drugs. ROS-mediated disruption of Δψ_m _constitutes a pivotal step in the apoptotic pathway of p53. Apoptosis triggered by p53 has been reported to be dependent on an increase in ROS and the release of apoptotic factors from mitochondrial damage [66,67]. These studies suggest that ROS are downstream mediators in p53-dependent apoptosis in transcription-dependent or transcription-independent pathways. When cells are exposed to oxidative stress, p53 is expressed at high levels by posttranslational modifications, including phosphorylation, acetylation, and glycosylation [68,69]. These events occur rapidly and lead to the activation of p53, resulting in either cell cycle arrest or apoptosis. These findings suggest the novel functions of ROS as p53 activators or p53 downstream effectors.

Studies outlined in this paper are highly significant because identification of a non-toxic dietary agent resveratrol that can delay onset and/or progression of prostate cancer could, in the long term, have a profound impact on the overall incidence of this malignancy. The capacity of resveratrol to promote killing of prostate tumor cells in response to TRAIL through induction of death receptors and activation of caspases in otherwise apoptosis-resistant cells suggests that resveratrol may induce fundamental alterations in cell signaling pathways leading to apoptosis.

## Conclusion

We have demonstrated that resveratrol induces apoptosis in prostate cancer cells through multiple mechanisms. It generates ROS, translocates p53 and Bax to mitochondria, regulates Bcl-2 family members and IAPs, and causes the release of mitochondrial proteins. Furthermore, our study establishes a direct role of p53 on the caspase-dependent mitochondrial death pathway and suggests that p53 interacts at the level of the mitochondria to influence resveratrol sensitivity. Gene therapy approach to deliver Smac/DIABLO can also be undertaken to sensitize TRAIL-resistant LNCaP cells. The ability of resveratrol to sensitize TRAIL-resistant LNCaP cells suggest that it can be combined with TRAIL for the prevention and/or treatment of prostate cancer.

## Methods

### Reagents

Anti-cytochrome c, anti-p53, and anti-Smac/DIABLO antibodies were purchased from BD Biosciences/Pharmingen (San Diego, CA). JC-1, and 5-(and-6)-chloromethyl-2',7'-dichlorodihydrofluorescein diacetate, acetyl ester (CM-H_2_DCFDA) were purchased from Invitrogen/Molecular Probes, Inc. (Eugene, OR). Antibodies against Bcl-2, Bcl-X_L_, Bax, Bak, PUMA, Noxa, Bim, Bid, AIF, survivin, XIAP, cIAP1, cIAP2 and β-actin were purchased from Santa Cruz Biotechnology Inc. (Santa Cruz, CA). Caspase-8 siRNA, Bax siRNA and control plasmids were purchased from BioVision, Inc. (Mountain View, CA). Enhanced chemiluminescence (ECL) Western blot detection reagents were from Amersham Life Sciences Inc. (Arlington Heights, IL). Smac siRNA and control plasmids were purchased from Imgenex (San Diego, CA). N-acetylcysteine (NAC), Terminal Deoxynucleotidyl Transferase Biotin-dUTP Nick End Labeling (TUNEL) assay kit, and caspase-3 and caspase-8 activity kits were purchased from EMD Biosciences/Calbiochem (San Diego, CA). Resveratrol was purchased from LKT Laboratories, Inc. (St. Paul, MN). Smac-N7 peptide (H-AVPIAQK-P-**RQIKIWFQNRRMKWKK**-OH) and control peptide (H-MKSDFYF-P-**RQIKIWFQNRRMKWKK**-OH) were modified to be cell permeable by linking the lysine carboxyl terminal to the arginine of *Antennapedia homeodomain *16-mer peptide (underlined) via a proline linker. TRAIL was produced according to published procedures [38].

### Cell Culture

LNCaP cells were obtained from the American Type Culture Collection (Manassas, VA) and cultured in RPMI 1640 supplemented with 10% fetal bovine serum (FBS) and 1% antibiotic-antimycotic (Invitrogen) at 37°C in a humidified atmosphere of 95% air and 5% CO_2_. Normal human prostate epithelial cells (PrECs) were purchased from Clonetics/Bio Whittaker (Walkersville, MD), and cultured in PrEBM medium (Cambrex, Rockland, ME).

### XTT Assay

Cells (1 × 10^4 ^in 200 μl culture medium per well) were seeded in 96-well plate and treated with or without drugs and incubated for various time points at 37°C and 5% CO_2_. Before the end of the experiment, 50 μl XTT labeling mixture (final concentration, 125 μM XTT (sodium 2,3-Bis(2-methoxy-4-nitro-5-sulfophenyl)-2H-tetrazolium-5-carboxanilide inner salt) and 25 μM PMS (phenazine methosulphate) per well was added and plates were incubated for further 4 h at 37°C and 5% CO_2_. The spectrophotometric absorbance of the sample was measured using a microtitre plate reader. The wavelength to measure absorbance of the formazon product was 450 nm, and the reference wavelength was 650 nm.

### Plasmids expressing dominant negative FADD and short hairpin RNA

Cells were plated in 60-mm dishes in RPMI 1640 containing 10% FBS and 1% penicillin-streptomycin mixture at a density of 1 × 10^6 ^cells/dish. The next day transfection mixtures were prepared. Cells were transfected with expression constructs encoding dominant negative FADD (DN-FADD) or empty vector in the presence of an expression vector pCMV-LacZ (Invitrogen life technologies) expressing β-galactosidase. For each transfection, 2 μg of DNA was diluted into 50 μl of medium without serum. After the addition of 3 μl of LipofectAMINE (Invitrogen life technologies) into 50 μl Opti-MEM medium, the transfection mixture was incubated for 10 min at room temperature. Cells were washed with serum-free medium, the transfection mixture was added, and cultures were incubated for 24 hrs in the incubator. The next day, culture medium was replaced with fresh RPMI 1640 containing 10% FBS and 1% penicillin-streptomycin mixture. Cells were treated with or without resveratrol. At the end of incubation, cells were harvested to measure protein expression by western blot analysis or apoptosis by DAPI staining.

For siRNA experiment, cells were transiently transfected with plasmids expressing caspase-8 siRNA (pGB-Caspase-8), Bax siRNA (pGB-Bax siRNA) or control siRNA (pGB-control) in the presence of pCMV-LacZ (Invitrogen life technologies) vector expressing β-galactosidase to control transfection efficiency. Cells were treated as described above.

### Soft Agar Assay

LNCaP cells (2 × 10^4 ^cells/well) were seeded in 12-well culture dishes in RPMI/0.35% bacto-agar over a bottom layer of RPMI/0.6% bacto-agar. Cells were then fed with growth media (100–200 μl/well) once a week until colonies grew to a suitable size for observation (about 3 weeks). Number of colonies were counted after they were stained with 3-(4,5-dimethylthiazol-2-yl)-2,5-diphenyltetrazolium bromide (1 mg/ml, 100 μl/well) overnight for better visualization.

### Apoptosis

Apoptosis was measured by the terminal deoxynucleotidyl transferase-mediated nick end-labeling method, which examines DNA strand breaks during apoptosis. Briefly, 1 × 10^5 ^cells were treated with curcumin at the indicated doses for various time points at 37°C. Thereafter, cells were washed with PBS, air-dried, fixed with 4% paraformaldehyde, and then permeabilized with 0.1% Triton ×-100 in 0.1% sodium citrate. After washing, cells were incubated with reaction mixture for 60 minutes at 37°C. Stained cells were mounted and analyzed under a fluorescence Olympus microscope (Olympus America Inc, Melville, NY). Pictures were captured using a Photometrics Coolsnap CF color camera (Olympus) and SPOT software (Diagnostic Instruments Inc., Sterling Heights, MI). Data were confirmed by staining cells with DAPI as previously described [41].

### Caspase Assay

Cells (3 × 10^4 ^per well) were seeded in a 96-well plate with 200 μl culture medium. Approximately 16 h later, cells were treated with various doses of resveratrol to induce apoptosis. Casapse-3 and caspase-8 activities were measured as per manufacturer's instructions (EMD Biosciences) with a fluorometer.

### Cellular Fractionation

Purified mitochondrial preparations were made as we described elsewhere [28]. In brief, cell pellets were resuspended in ice-cold buffer A (250 mM sucrose, 20 mM HEPES, 10 mM KCl, 1.5 mM MgCl_2_, 1 mM EDTA, 1 mM EGTA, 1 mM DTT, 17 μg/ml phenylmethylsulfonyl fluoride, 8 μg/ml aprotinin, 2 μg/ml leupeptin, pH 7.4]. Cells were homogenize with either 20 strokes of a Dounce homogenizer or 23-gauge needles on ice, and the suspension was centrifuged at 750 × g for 10 min at 4°C to remove nuclei. The supernatant was spun and the resulting mitochondrial pellets were layered over a 1 to 2 mM sucrose step gradient [10 mM Tris (pH 7.6), 5 mM EDTA, 2 mM DTT, and 1 × protease inhibitor cocktail) and centrifuged at 22,000 × g for 30 min at 4°C. Mitochondria were collected at the 1 to 1.5 M interphase. The supernatant from the previous step was spun to obtain the cytoplasmic S100 fraction. The protein concentrations were determined by Bradford method (Bio-Rad, Hercules, CA). The purification of S-100 protein was determined by Western blot analysis using anti-cytochrome oxidase IV antibody.

### Western Blot Analysis

Cell pellets were lysed in RIPA buffer containing 1 × protease inhibitor cocktail, and protein concentrations were determined using the Bradford assay (Bio-Rad, Philadelphia, PA). Cell lysates were electrophoresed in 12.5% SDS polyacrylamide gels and then transferred onto nitrocellulose membranes. After blotting in 5% nonfat dry milk in TBS, the membranes were incubated with primary antibodies at 1:1,000 dilution in TBS-Tween 20 overnight at 4°C, and then secondary antibodies conjugated with horseradish peroxidase at 1:5,000 dilution in TBS-Tween 20 for 1 hour at room temperature. Protein bands were visualized on X-ray film using an enhanced chemiluminescence system.

### Measurement of Mitochondrial Membrane Potential (ΔΨ_m_)

Mitochondrial membrane potential was measured as we described elsewhere [28]. Mitochondrial energization was determined by retention of JC-1 dye (Molecular Probes Inc., Eugene) as we described earlier [28,40]. Briefly, drug treated cells (5 × 10^5^) were loaded with JC-1 dye (1 μg/ml) during the last 30 min of incubation at 37 °C in a 5% CO_2 _incubator. Cells were washed in PBS twice. Fluorescence was monitored in a fluorometer using 570-nm excitation/595-nm emission for the J-aggregate of JC1 [70]. ΔΨ_m _was calculated as a ratio of the fluorescence of J-aggregate (aqueous phase) and monomer (membrane-bound) forms of JC1.

### Determination of Reactive Oxygen Species (ROS)

ROS was measured as we described elsewhere [53]. In brief, LNCaP cells were seeded in 96-well plates. After 16 h, cells were loaded with 5 μM CM-H_2_DCFDA dye for 30 min, and treated with either curcumin (10 or 20 μM) or 0.05% DMSO for 0–360 min. Fluorescence was measured at excitation wavelength of 488 nm and emission wavelength of 515 nm using a fluorescence plate reader.

### Immunocytochemistry

Cells were grown on fibronectin-coated coverslips (Beckton Dickinson, Bedford, MA), washed in PBS, and fixed for 15 min in 4% paraformaldehyde. Cells were permeabilized in 0.1% Triton ×-100, washed and blocked in 10% normal goat serum. Cells were incubated with anti-cytochrome c, anti-Smac, anti-Bax or anti-p53 antibody (1:200) for 18 h at 4°C. Cells were then washed and incubated with fluorescently labeled secondary antibodies (1:200) along with DAPI (1 μg/ml) and mitotracker red for 1 h at room temperature. Cells were washed and coverslips were mounted using Vectashield (Vector Laboratories, Burlington, CA). Isotype-specific negative controls were included with each staining. Stained cells were mounted and visualized under a fluorescence Olympus microscope (Olympus America Inc.). Pictures were captured using a Photometrics Coolsnap CF color camera (Olympus) and SPOT software (Diagnostic Instruments Inc.).

### Statistical Analysis

All data were presented as mean ± SD from at least three sets of independent experiments. ANOVA analysis with Turkey's multiple comparisons was used to determine the significance of statistical differences between data at the level of *P *< 0.05.
